# Role of Oxidative Stress in the Pathology and Management of Human Tuberculosis

**DOI:** 10.1155/2018/7695364

**Published:** 2018-10-11

**Authors:** Madhur D. Shastri, Shakti Dhar Shukla, Wai Chin Chong, Kamal Dua, Gregory M. Peterson, Rahul P. Patel, Philip M. Hansbro, Rajaraman Eri, Ronan F. O'Toole

**Affiliations:** ^1^School of Health Sciences, College of Health and Medicine, University of Tasmania, Launceston, Australia; ^2^Priority Research Centre for Healthy Lungs, School of Biomedical Sciences and Pharmacy, The University of Newcastle, Callaghan, Australia; ^3^Department of Molecular and Translational Science, Monash University, Clayton, Australia; ^4^Discipline of Pharmacy, Graduate School of Health, University of Technology Sydney, Ultimo, NSW, Australia; ^5^Pharmacy, College of Health and Medicine, University of Tasmania, Hobart, Australia; ^6^School of Medicine, College of Health and Medicine, University of Tasmania, Hobart, Tasmania, Australia; ^7^Department of Clinical Microbiology, Trinity College Dublin, Dublin, Ireland

## Abstract

Tuberculosis (TB), caused by the bacterium *Mycobacterium tuberculosis*, is the leading cause of mortality worldwide due to a single infectious agent. The pathogen spreads primarily via aerosols and especially infects the alveolar macrophages in the lungs. The lung has evolved various biological mechanisms, including oxidative stress (OS) responses, to counteract TB infection. *M. tuberculosis* infection triggers the generation of reactive oxygen species by host phagocytic cells (primarily macrophages). The development of resistance to commonly prescribed antibiotics poses a challenge to treat TB; this commonly manifests as multidrug resistant tuberculosis (MDR-TB). OS and antioxidant defense mechanisms play key roles during TB infection and treatment. For instance, several established first-/second-line antitubercle antibiotics are administered in an inactive form and subsequently transformed into their active form by components of the OS responses of both host (nitric oxide, *S*-oxidation) and pathogen (catalase/peroxidase enzyme, EthA). Additionally, *M. tuberculosis* has developed mechanisms to survive high OS burden in the host, including the increased bacterial NADH/NAD^+^ ratio and enhanced intracellular survival (Eis) protein, peroxiredoxin, superoxide dismutases, and catalases. Here, we review the interplay between lung OS and its effects on both activation of antitubercle antibiotics and the strategies employed by *M. tuberculosis* that are essential for survival of both drug-susceptible and drug-resistant bacterial subtypes. We then outline potential new therapies that are based on combining standard antitubercular antibiotics with adjuvant agents that could limit the ability of *M. tuberculosis* to counter the host's OS response.

## 1. Introduction to Tuberculosis

Tuberculosis (TB) is the leading cause of mortality worldwide due to a single infectious agent, killing approximately 1.67 million people in 2016 alone [1, 2]. There were an estimated 10.4 million new cases of the disease globally in 2016, corresponding to an annual incidence rate of approximately 140 per 100,000 persons [1]. The highest incidence rates of TB were seen in South Africa (781/100,000), Lesotho (724/100,000), and the Philippines (554/100,000) in 2016 [1]. Five countries recorded more than 0.5 million new cases nationally and accounted for >50% of the global total in 2016, i.e., India (2,790,000 cases), Indonesia (1,020,000 cases), China (895,000 cases), Philippines (573,000 cases), and Pakistan (518,000 cases) [1].

The World Health Organization (WHO) has set End TB Strategy targets to reduce global TB mortality by 95% and the incidence of TB by 90% to less than 10 cases per 100,000 by 2035, with respect to 2015 levels [3]. While industrialised countries such as Australia are considered to have a low TB burden (incidence rate of 5.7/100,000 nationally in 2014 [4]), the WHO has also established specific targets for low TB incidence countries for the pre-elimination of TB by 2035 (defined as <10 TB cases per million population) and the elimination of TB by 2050 (<1 TB case per million population) [5]. Reaching targets for the elimination of TB will require major advances in our understanding of the biology of the disease, as well as improvements in the effectiveness and accessibility of preventive, diagnostic, and therapeutic interventions.

## 2. Pathogenesis of Tuberculosis

TB in humans is primarily caused by the bacterial species, *Mycobacterium tuberculosis*, but can also be caused by other members of the *M. tuberculosis* complex (MTBC), including *M. africanum* and *M. bovis*. It is primarily spread via the release of aerosols from the lungs of patients with active TB through coughing, sneezing, or spitting. Infection of a new host commences following inhalation of droplets containing the pathogen deep into the air sacs of the lungs. There, the bacterium becomes internalized by resident alveolar macrophages. *M. tuberculosis* is able to prevent phagosome maturation and, hence, fusion with lysosomes [6, 7], which enables the pathogen to persist inside the macrophage [8]. At this stage, active TB is not inevitable. In most individuals who become infected with *M. tuberculosis*, a latent infection ensues whereby bacteria are maintained in a low replicative, but viable, state in the lung with minimal clinical signs or symptoms evident [9, 10].

In 2016, Houben and Dodd determined the proportion of the world's population that are latently infected with TB to be 23.0% (95% uncertainty interval [UI]: 20.4%–26.4%), or approximately 1.7 billion people [11]. However, the high-carriage rate provides a reservoir for subsequent disease in susceptible hosts whereby the age-weighted lifetime risk of the development of clinical TB in a latently infected individual has been estimated to be 12% [12]. Furthermore, large numbers of people develop active TB every year following recent close contact with an index TB case [13].

If the infected macrophage is unable to contain the replication of *M. tuberculosis*, a granulomatous inflammatory disease results. Here, additional macrophages surround the infected macrophage and fuse to generate a multinucleated giant cell [14]. Mature human tubercular lesions have been found to consist of a central zone of caseous necrotic tissue bordered by T lymphocytes and macrophages and an area that also includes aggregates of B cells [14]. The ongoing inflammation can lead to a remodelling of the lung architecture, which manifests as extensive fibrosis, cavitation, traction bronchiectasis, bronchostenosis, or parenchymal lung destruction [15, 16]. The pathology caused by TB results in significant lung function impairment as measured by a forced expiratory volume in 1 second (FEV_1_) < 60% predicted at diagnosis [17]. In addition to dyspnea, additional symptoms of active TB include fever, night sweats, and weight loss. Without appropriate chemotherapy, untreated smear-positive TB in HIV-negative individuals has a 10-year case fatality between 53% and 86%, with a weighted mean of 70% [18].

Even after successful adherence to and completion of TB treatment, former patients may develop long-term airflow obstruction and a restrictive loss of pulmonary function. A large, international population-based study, Burden of Obstructive Lung Disease (BOLD), investigated the association of airflow obstruction and spirometric restriction with a history of TB. Examining a study population of 14,050 participants from 18 countries, the study established that a self-reported history of TB was significantly associated with both airflow obstruction (adjusted OR of 2.51 [95% CI: 1.83–3.42]) and spirometric restriction (adjusted OR of 2.13 [95% CI: 1.42–3.19]) [19]. The authors of the BOLD study concluded that a history of TB “should be considered as a potentially important cause of obstructive disease and low lung function, particularly where TB is common” [19]. Therefore, identification of the specific mechanisms and events that lead to lung tissue damage in TB patients is essential for tempering the impact of the disease.

## 3. Effect of Oxidative Stress on the Lungs

Oxidative stress (OS) arises due to an imbalance between the free reactive oxygen species (ROS) and the antioxidant mechanisms [20, 21]. In the lung, there is a higher risk of OS compared to other organs [22]. Under physiological conditions, the lung is exposed to approximately 10–15,000 litres of air every day, each breath containing a myriad of exogenous oxidative compounds, such as pollutants, tobacco smoke, and allergens [23]. All of these exogenous factors stimulate inflammatory cells to generate free radicals [20, 24]. Moreover, under these circumstances, enzyme reaction pathways, such as nicotinamide adenine dinucleotide phosphate oxidases, myeloperoxidase, xanthine oxidase, and eosinophil peroxidase, are activated to produce endogenous ROS, including hydrogen peroxide, hydroxyl radical, and superoxide radical [24]. In order to compensate this burden, the lung has evolved numerous antioxidant defense mechanisms.

There are two distinct groups of antioxidant processes: enzymatic and nonenzymatic systems. Enzymatic antioxidant processes present in the lung include superoxide dismutases (SOD), glutathione peroxidase, and catalase, whereas nonenzymatic processes involve ferritin, ascorbic acid ceruloplasmin, and carotene [24–26]. Together, these antioxidant mechanisms buffer oxidants and maintain the oxidative balance in the lung. However, it is important to note that such complex antioxidant mechanisms can be overwhelmed if the production of ROS is greater than the capacity of cells to scavenge it, leading to OS [20, 24].

Excess formation of ROS can initiate series of chemical reactions and cause damage to cellular components, including proteins, lipids, and nucleic acids [20, 24, 25]. Furthermore, ROS can also initiate inflammatory signaling cascades via protein kinase pathways, transcription factors, and genomic expression of proinflammatory regulators, leading to an overactivated immune system [27]. Numerous studies have linked OS to various lung disorders, including asthma, chronic obstructive pulmonary disease (COPD), acute pulmonary distress syndrome, and TB [28, 29]. For instance, airway smooth muscle cells isolated from patients with COPD had elevated ROS levels compared to healthy individuals [30]. Likewise, asthmatic patients had higher levels of oxidant products, including peroxidase, chlorotyrosine, malondialdehyde, and bromotyrosine [28]. Patients suffering from acute respiratory distress syndrome exhibit an oxidant : antioxidant imbalance, as well as high levels of peroxidase and oxidized *α*1-antitrypsin [28].

## 4. Oxidative Stress Response against *Mycobacterium tuberculosis* Infection

It is noteworthy that OS is a double-edged sword and proceeds in an indiscriminate chemical manner. Although ROS can damage the host cells, it also kills infectious agents, including invading pathogens within the host. At the molecular level, excess OS causes damage to foreign pathogens via direct binding of ROS to amino-acid side chains like arginine, threonine, lysine, and proline and/or histidine oxidation or modification of tyrosine to nitrotyrosine, affecting the structural and functional properties of proteins [31]. ROS is highly toxic to bacteria as it can either directly destroy DNA, protein, and lipids or indirectly damage the nucleic acid via oxidation of the nucleotide pool [32]. Such damage is reported to be due to the interaction of ROS and bacteria via various biochemical processes, including the Fenton reaction and the tricarboxylic acid cycle [32].

Furthermore, excess ROS production can trigger a series of oxidative reactions on polyunsaturated fatty acids present on the cell membrane of microorganism(s), collectively known as lipid peroxidation [31]. These reactions can critically destabilise and impair microorganisms' homeostasis [31]. Hence, the human body generates a balanced amount of ROS as an antimicrobial defense arsenal against pathogens. For example, innate immune cells, such as neutrophils, employ an immune process known as respiratory burst as an antimicrobial defense against Candidiasis by rapid production and release of ROS onto the invading *Candida albicans* [33].

*M. tuberculosis* invades and replicates within the host macrophages. As an immune response, the infected macrophage initiates a respiratory burst and produces high levels of ROS to counteract and kill the mycobacteria [34]. Upregulated serum levels of malondialdehyde (an indicator for lipid peroxidation) are observed in patients with *M. tuberculosis* disease compared to healthy controls [34]. It is important to point out that the survivability of *M. tuberculosis* is highly dependent on the levels of ROS produced by the host immune cells [35]. If the ROS levels are overwhelmed by *M. tuberculosis* antioxidant systems, then the pathogen will continue to survive and replicate in the host [35].

Mechanistically, among all the ROS, nitric oxide (NO) is known to be one of the major contributors as an anti-TB agent. NO is synthesized by NO synthase in macrophages, and NO synthase-deficient mice have been shown to exhibit increased susceptibility to *M. tuberculosis* [36]. Patients with active pulmonary TB have higher levels of NO in their lungs [36]. The accumulation of ROS during OS can further produce superoxide, hydrogen peroxide, and hydroxyl radicals via single-electron reduction chain reactions [32]. Although studies have reported that *M. tuberculosis* possesses bacterial SODs and catalases that are known to degrade superoxide and hydrogen peroxide, respectively, there is no countermechanism for hydroxyl radicals [32]. Interestingly, *M. tuberculosis* responds to ROS in a concentration-dependent manner. Exposure of *M. tuberculosis* to a low concentration of ROS (0–5.0 mM hydrogen peroxide) had no effect on the bacterial cell viability, but it initiated the expression of responsive genes which are sensitive to ROS [37], whereas high concentrations of ROS (50–200 mM hydrogen peroxide) were found to be lethal to *M. tuberculosis* cells [37]. Collectively, when the levels of ROS produced by host cells are low, *M. tuberculosis* triggers the release of DNA damage-responsive genes that initiate DNA repair mechanisms within the bacteria to counteract the damage done by ROS [37, 38]. However, such counteractive mechanisms of *M. tuberculosis* do not protect against high levels of ROS [37, 38].

## 5. Chemotherapy of Tuberculosis

The standard therapeutic regimen for drug-susceptible pulmonary TB recommended by the WHO requires patients to take four drugs: isoniazid, rifampicin, pyrazinamide, and ethambutol for 2 months, followed by continuation with isoniazid and rifampicin for an additional 4 months (2HRZE/4HR) [39]. This is a lengthy treatment duration for a bacterial disease, and clinical trials are in progress to identify an effective, shortened regimen. This includes the bedaquiline, pretomanid, moxifloxacin, and pyrazinamide (BPaMZ) regimen and the bedaquiline, pretomanid, and linezolid (BPaL) combination, which are currently in advanced phase 2 clinical trials through the TB Alliance (https://www.tballiance.org/).

Another driver for the development of new regimens to manage TB is the emergence of MDR-TB that are resistant to the key first-line drugs, isoniazid and rifampicin. There was an approximate 95% increase in estimated MDR-TB cases from 250,000 per year in 2009 to 490,000 cases per year in 2016 [40]. In addition, there were an additional 110,000 cases that were susceptible to isoniazid but resistant to rifampicin in 2016 [1]. Countries with the highest proportion of new TB cases being MDR are located in the WHO European region, where an estimated 71,000 incident cases of rifampicin-resistant TB and MDR-TB emerged in 2016 [41]. For example, in Belarus, 38% of new TB cases and 72% of previously treated cases in 2016 were MDR [1]. This is followed closely by the Russian Federation, where 27% of new TB cases and 65% of previously treated cases were MDR in the same period [1].

MDR-TB is difficult and costly to treat. The treatment completion success rate drops from 83% overall for TB to 54% for MDR-TB cases globally due to a higher frequency of treatment failure, loss to follow-up, and death of patients [1]. Furthermore, the estimated costs associated with treating a case of TB increase substantially when going from drug-susceptible TB (USD $17,000 in the USA, €10,282 in 15 European Union (EU) countries, per case) to MDR-TB (USD $134,000 in the USA, €57,213 in 15 EU countries, per case) [42, 43]. The standard treatment for MDR-TB recommended by the WHO has consisted of an intensive 8-month phase with at least five effective TB drugs, which included pyrazinamide (group D1) and at least four core second-line drugs: one from group A, one from group B, and at least two drugs from group C (Table 1). When the drug-susceptibility profile of the isolate or drug toxicity in the patient prevents the minimum number of five effective TB drugs, drugs from group D2 and D3 are added to bring the total to five [44]. This is then followed by a continuation phase, with at least three of the second-line anti-TB drugs that are most potent against the specific patient isolate of *M. tuberculosis*, for a total treatment duration of 20 months [44]. In 2016, the WHO updated its treatment guidelines and recommended a shorter 9- to 12-month treatment duration for MDR-TB based on its successful use in Bangladesh and several other countries [45]. Here, the regimen consists of a 4- to 6-month intensive phase with seven drugs, i.e., moxifloxacin (group A), kanamycin (group B), prothionamide (group C), clofazimine (group C), pyrazinamide (group D1), high-dose isoniazid (group D1), and ethambutol (group D1), followed by a 5-month continuation phase with moxifloxacin, clofazimine, pyrazinamide, and ethambutol [45]. This newer regimen decreases the cost of MDR-TB treatment and is expected to reduce patient loss from therapy.

TB treatment is associated with levels of toxicity in patients, in some cases, requiring discontinuation of specific drugs. The first-line drugs isoniazid and pyrazinamide are associated with hepatotoxicity [46]. Along with rifampicin, they are also associated with gastrointestinal upset and rash [46]. Ethambutol, on the other hand, can cause ocular toxicity [47, 48]. In terms of second-line TB drugs, moxifloxacin and other fluoroquinolones carry a risk of lengthening the QT interval in the heart's electrical cycle [49]. Kanamycin and other second-line aminoglycosides, which require parenteral injection, are associated with hearing loss, nephrotoxicity, skin rash, hypersensitivity, and peripheral neuropathy [49]. Prothionamide causes gastrointestinal disturbance, including vomiting, while clofazimine can lead to skin discolouration and QT prolongation [49]. It is therefore apparent that there is still a need to improve the therapeutic management of TB—to increase the efficacy of TB drugs to further reduce the duration of treatment and to minimise adverse side effects for patients.

## 6. Role of Oxidative Processes in the Activation of TB Prodrugs

Several of the TB drugs are administered to patients in an inactive form and must be activated inside the host. One of those is isoniazid (isonicotinylhydrazide). This prodrug is oxidatively activated by a catalase/peroxidase enzyme present in *M. tuberculosis*, KatG, to produce an isonicotinic acyl radical [50]. This couples with NAD^+^ and NADP^+^ to form a number of adducts which inhibit a 2-trans-enoyl-acyl carrier protein reductase, InhA, an essential component in mycolic acid biosynthesis [50]. Conversely, *M. tuberculosis* is particularly sensitive to OS and hence requires KatG for protection against ROS, including hydrogen peroxide. Paradoxically, then, in shielding the bacterial cell from the toxic activities of ROS, KatG also exposes the pathogen to the anti-infective action of isoniazid [51].

Pretomanid, a bicyclic nitroimidazole, is a prodrug that requires activation by a deazaflavin- (cofactor F420-) dependent nitroreductase (Ddn) to produce three primary metabolites, with des-nitroimidazole (des-nitro) being the major antimycobacterial component [52]. The des-nitro metabolite generates reactive nitrogen species, including NO, and these provide anaerobic killing of *M. tuberculosis* cells [53]. Hence, using a bacterial-encoded enzyme to activate the prodrug, pretomanid, creates intracellular levels of nitrosative stress which are toxic to *M. tuberculosis*. The thioamides, ethionamide, and its analogue, prothionamide, are also prodrugs and are activated by an enzyme, EthA, encoded by *M. tuberculosis*. EthA is a flavoprotein monooxygenase that catalyzes the NADPH- and O_2_-dependent monooxygenation of thioamides [54]. The metabolites generated target InhA and inhibit mycolic acid synthesis in a similar manner to isoniazid [55].

## 7. Mechanisms of Oxidative Stress Tolerance and Drug Resistance in *Mycobacterium tuberculosis*

**Figure 1:**
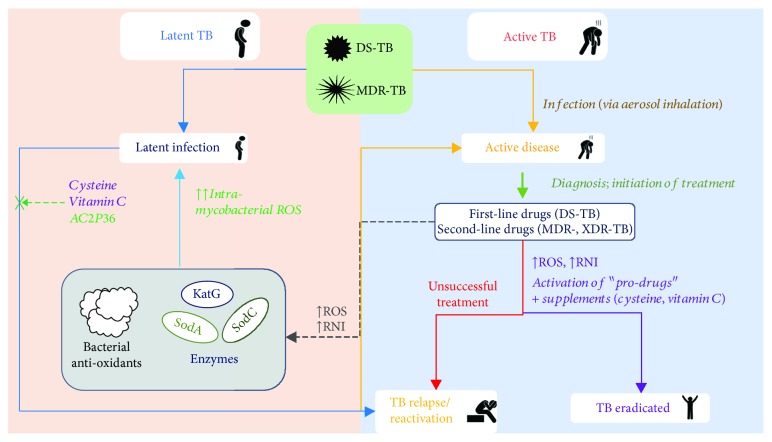
Interplay of oxidative stress and tuberculosis. Infection with drug-susceptible or MDR-TB can lead to active clinical disease in humans prompting diagnosis and the prescription of first- or second-line drugs, respectively. TB disease also leads to increased oxidative burden (ROS, RNI) in the lung that aids in the activation of TB prodrugs. Supplements such as vitamin C may augment the treatment of TB by inhibiting the emergence of “persisters” through mechanisms including downregulation of mycobacterial siderophore biosynthesis. Unsuccessful treatment can lead to subsequent TB relapse. Mycobacterium tuberculosis can also remain dormant in the host during a latent TB infection. Administration of supplements (cysteine, vitamin C, and AC2P36) may provide a pathway for inhibiting the reactivation of latent TB potentially by increasing the intramycobacterial OS and alteration in “lipid/thiol” biosynthesis.

It is now known that *M. tuberculosis* shows marked survival within the host despite the toxic effects of ROS, in particular due to its ability to effectively monitor and counter redox signals through a number of mechanisms. The interplay between oxidative stress and TB is depicted in Figure 1. Several studies have implicated a key role of cell wall-associated lipids (mycolic acids) in forming a physical barrier to counter host-generated exogenous OS. Utilising a combination of lipidomic and genomic approaches, Portevin et al. reported significant differences in the proportion of structural variants of mycolic acids within and between phylogenetic lineages of *M. tuberculosis* [56]. The presence of differences in mycolic acids in the cell wall could affect the interaction of *M. tuberculosis* with the host cells [56].

Furthermore, *M. tuberculosis* has also developed several cellular mechanisms to counter the redox imbalance that may also adversely affect the efficacy of frontline antitubercle antibiotics. For instance, an increase in the mycobacterial NADH/NAD^+^ ratio, through mutation of *ndh* which encodes a type II NADH dehydrogenase, confers coresistance to isoniazid and ethionamide [57]. *M. tuberculosis* strains that exhibit resistance to isoniazid and pretomanid also demonstrate higher resistance to ROS compared to wild-type *M. tuberculosis* (CDC1551 and CB3.3) [34, 36, 58]. These strains were also found to have increased resistance towards peroxide and acidified nitrite [35, 58]. Several strains of *M. tuberculosis* are equipped to produce a specific protein, namely, enhanced intracellular survival (Eis), which can detect ROS and respond in a counteractive manner [59]. Moreover, *M. tuberculosis* possesses proteasome (peroxiredoxin) that can recognize, repair, and remove oxidative altered or damaged proteins [60]. More recently, another peroxiredoxin system, comprised of NADPH, thioredoxin reductase (TrxR), and thioredoxin, has been shown to be highly effective in protecting *M. tuberculosis* against ROS stress [60]. *M. tuberculosis* utilizes the thioredoxin (Trx) system for defense against OS through its disulfide reductase activity regulating proteins' dithiol/disulfide balance [61]. Betts et al. documented that dormant *M. tuberculosis* under nutrient-limiting conditions exhibit arrested growth, reduced respiration rate, and increased tolerance against isoniazid, rifampicin, and metronidazole [62].

*M. tuberculosis* also expresses alternative peroxidase systems to maintain its virulence, including the expression of alkyl hydroperoxide reductase subunit C (AhpC) [63]. AhpC relates to the peroxiredoxin family and contains three cysteine residues in its active site and has been shown to be overexpressed in *M. tuberculosis* exhibiting resistance to isoniazid [64]. Mycobacteria utilize AhpC to detoxify organic peroxides by reduction into less reactive alcohol derivatives. Master et al. demonstrated that AhpC plays an important role in the early stages of reactivation of *M. tuberculosis* and/or upon transmission of *M. tuberculosis* to a new host [65].

*M. tuberculosis* also generates and secretes antioxidant enzymes to persist in the abnormal redox environment; these enzymes include SOD and KatG. Notably, *M. tuberculosis* harbours one catalase (KatG) gene that accounts for catalase, peroxidase, and peroxinitritase activity [66]. KatG is also a known virulence factor in *M. tuberculosis* as it aids bacterium to persist within the infected host tissues [67]. In addition to KatG, *M. tuberculosis* also contains two metalloenzymes, an iron-containing SOD (SodA or FeSOD) and a copper- and zinc-containing SOD (SodC or CuZnSOD) [68, 69]. SodA is expressed constitutively under homeostatic conditions and is a major secretory protein of *M. tuberculosis*, which has been shown to impart protection against superoxide in *in vitro* experiments [70]. The expression of SodA is enhanced by hydrogen peroxide exposure and nutrient starvation [62]. SodC constitutes only a minor proportion of mycobacterial superoxide dismutase activity; however, SodC contains a lipoprotein-binding site, suggesting that it is anchored on the cell wall, and has been demonstrated to protect the bacterium from ROS [71].

Other recent advances have been made to understand how *M. tuberculosis* survives an oxidative burden in the host. For instance, Nambi et al. performed genome-wide genetic interaction assays and demonstrated that SodA forms a membrane-associated oxidoreductase complex with DoxX (a membrane protein) and SseA (thiol oxidoreductase). This SodA-DoxX-SseA complex links radical detoxification with cytosolic thiol homeostasis, and any abnormality in this complex results in defective recycling of mycothiol and accumulation of cellular oxidative damage [29]. Overall, coordination between oxygen radical detoxification and thiol homeostasis is required for *M. tuberculosis* to encounter and overcome the oxidative environment in the host.

Genomic analysis has revealed the presence of heme-based redox sensors (DosS and DosT) in *M. tuberculosis*, which has been reviewed elsewhere [72]. Kumar et al. reported that DosS is rapidly autooxidized to met (Fe_3_^+^) form, whereas DosT exists in the O_2_-bound (oxy) form [73]. Notably, O_2_, NO, and CO act as ligands for both DosS and DosT [73]. The authors also proposed that DosS acts as a redox sensor and DosT is primarily a hypoxia sensor [73]. It has been also reported that both DosS and DosT activate transcriptional regulator DosR, leading to the induction of the DosR regulon that aids the anaerobic survival of *M. tuberculosis* and potentially leads to latent infection [74]. DosR regulon plays a critical role in the survival of *M. tuberculosis* in the oxygen-depleted microenvironment (during anaerobic dormancy), as well as rapid recovery of bacterial reproducibility once the favorable growth conditions prevail [75]. Mechanistically, Yang et al. have demonstrated that deacetylation of DosR enhances its DNA-binding ability and promotes the transcription of target genes (primarily flavin adenine dinucleotide binding, oxidoreductase activity, and coenzyme binding), thus allowing *M. tuberculosis* to shift to dormancy under hypoxia [76].

## 8. Potential New TB Therapies Targeting the Oxidant : Antioxidant Balance

As mentioned, several front-line TB drugs need to be activated first to demonstrate their complete antitubercle properties [57, 62]. The activation of these antimycobacterial prodrugs is achieved by the action of oxidants released from the host immune cells [77]. However, the evolution of bacterial capability to neutralize the host's OS has also led to the nonactivation of these “inactivated” form of drugs to exert their effects on the pathogen. Therefore, it is necessary to investigate and develop drugs with the potential to limit the ability of *M. tuberculosis* to counter the host's OS, simultaneously enhancing the activation of prodrugs and elimination of bacterium.

Interestingly, targeting cytochrome P450 enzymes in *M. tuberculosis* has also been proposed as the source of a potential new class of drugs for treating MDR-TB [78]. Similarly, targeting oxidative phosphorylation (NADH dehydrogenase, menaquinone biosynthesis, terminal oxidase, and ATP synthase) with specific inhibitors (phenothiazine derivatives, DG70, imidazopyridine amides, and diarylquinolines), in combination with antitubercle drugs, is an exciting novel avenue for short-term and effective treatment [79]. Notably, inhibiting bacterial oxidative phosphorylation may also result in limiting the formation of “persisters” in the host.

Treatment with cysteine (in a concentration- and time-dependent manner with increased oxygen consumption) has recently been shown to prevent the emergence of “persisters” and isoniazid resistance by increasing the respiration rates and thus promoting both OS and metabolism in the pathogen [80]. Similar results were observed with rifampicin. In infected murine macrophages, the combination of isoniazid and N-acetylcysteine (NAC) was found to be significantly more effective in eliminating intracellular *M. tuberculosis* than isoniazid alone [80]. *In vitro* experiments revealed that cysteine (when added within 1 day of isoniazid treatment) caused increasing delays in the growth of isoniazid-resistant mutants, which was completely inhibited at highest concentration (4 mM). Interestingly, both isoniazid and the combination of isoniazid and cysteine resulted in similar bacterial killing in a strain-independent manner (Mtb H37Rv, Mtb Beijing, and Mtb CDC1551) for the first 7 days; however, only isoniazid/cysteine combination yielded approximately 6-log killing of the initial cultures after 3 weeks, without development of isoniazid-resistant strains. Thus, the use of supplements in combination with antitubercle drugs may prevent the formation of “persister” bacteria, as well as producing more effective killing of *M. tuberculosis* in shorter time periods [81].

Several other potential combinatorial therapeutic strategies are now being investigated. Vilchèze et al. reported that vitamin C (4 mM) specifically sterilizes *in vitro* cultures of *M. tuberculosis*, including drug-susceptible (H37Rv) and MDR-resistant (mc^2^4997) and extensively drug-resistant (TF275) variants of bacillus. The transcriptional profile of vitamin C-treated *M. tuberculosis* revealed downregulation in the *mbtD* gene (biosynthesis of siderophore) [82]. The antitubercle activity of vitamin C depends upon the high concentration of ferrous ion leading to increased free ions levels, both intracellularly (50–75%) and extracellularly (4-fold). Vitamin C treatment (4 mM) also increased ROS (superoxide, hydrogen peroxide, and hydroxyl radicals) production (~3-fold) via the Haber-Weiss and Fenton reactions, which can result in DNA fragmentation measured by the terminal deoxynucleotidyl transferase-mediated dUTP-biotin nick end labelling (TUNEL) assay [82]. The effects of vitamin C were not observed when culturing *M. tuberculosis* in anaerobic conditions (oxygen level < 0.001%), suggesting a prooxidant effect of vitamin C [82]. In addition, mycothiol-deficient strain of *M. tuberculosis* (ΔmshA), hypersensitive to OS, showed rapid growth reduction in the presence of vitamin C, confirming the role of the oxidative process in its bactericidal activity [82]. Vitamin C treatment also affected the FASI (eukaryotic-like fatty acid synthase type I) potentially leading to altered lipid biosynthesis in an NADPH concentration-dependent manner. Collectively, vitamin C exhibits bactericidal activity against *M. tuberculosis* most likely due to its prooxidant activity, at least in *in vitro* cultures [82].

Recently, Vilchèze et al. have also shown that addition of vitamin C (1 mM) to isoniazid/rifampicin sterilized the *in vitro* cultures of drug-susceptible *M. tuberculosis* more rapidly than the drugs alone (~1 week). Similar effects were observed with even second-line TB drugs, namely, fluoroquinolone ofloxacin (OF), the injectable kanamycin (Km), and ETH [83], where addition of vitamin C reduced the sterilization time by 9 days compared to drugs alone [83]. Moreover, the authors also demonstrated that combining vitamin C with isoniazid and rifampin reduced the bacterial burden in the lungs of mice infected with *M. tuberculosis* over a shorter time frame than isoniazid and rifampin combination only [83]. Briefly, buffered aqueous 1.7 M solution (3 g/kg) of vitamin C was intraperitoneally administered to CBA/J female mice and infected with either low (28 CFUs) or high (105 CFUs) doses of *M. tuberculosis* (H37Rv) via the aerosol route. The mice receiving lower infective dose and treated with the combination of isoniazid/rifampicin/vitamin C exhibited lower lung bacterial burden (~one log) compared to mice treated with isoniazid/rifampicin in average at four weeks post-infection. Post-6-week treatment, *M. tuberculosis* was recovered from the lungs of one mouse in both isoniazid/rifampicin/vitamin C and isoniazid/rifampicin groups. The mice receiving higher infective dose showed a significantly lower bacterial recovery from lung only after 6 weeks of treatment in the isoniazid/rifampicin/vitamin C group [83]. The studies on the efficacy of vitamin C supplementation in TB outcomes have been largely positive (both in humans and animal models); however, focused studies addressing several pointed questions (vitamin C quantity, spectrum of activity, etc.) in clinical trials could provide definitive rationale and the underlying mechanism of action for the treatment of TB [84].

Another compound, AC2P36 (5-chloro-N-(3-chloro-4-methoxyphenyl)-2-methylsulfonylpyrimidine-4-carboxamide), has been shown to kill *M. tuberculosis* by directly depleting the amount of free thiols, especially at acidic pH (5.7), and to enhance the bactericidal activity of commonly used antitubercle drugs, including isoniazid and clofazimine [85]. The antitubercle activity of AC2P36 seems to be associated with “thiol stress,” which leads to increased accumulation of intracellular ROS, at acidic pH [85].

Collectively, these studies highlight the crucial roles of oxidant : antioxidant balance in limiting and treating *M. tuberculosis* infections. The utilization of relatively safe and inexpensive therapeutic supplements, such as vitamin C or cysteine, may aid in bacterial killing through increasing ROS burden in the bacterium and alterations in the lipid/thiol biosynthesis in a shorter time frame and without the formation of a drug-resistant “persister” population in the host. Future studies should also focus on developing novel strategies to target the DosS/DosT mechanisms as well as mycolic acid biosynthesis for *M. tuberculosis* survival, in combination with existing antibiotics that may pave the way for improved therapies to treat the disease.

## 9. Conclusions

Oxidative stress mediated by host cells, especially macrophages, plays a pivotal role to prevent the vicious cycle of *M. tuberculosis*. However, certain strains of *M. tuberculosis* are known to be resistant against the inhibitory effects of the oxidative burst produced by macrophages, primarily via the presence of lipids, proteins, enzymes, and antioxidant defense systems, like SOD, KatG, and DosR regulon, within the mycobacteria. These act in a counteractive mode against OS. These antioxidative mechanisms also affect the antibiotic drug treatment. Various research findings have led to a greater understanding of the effects of both drug-susceptible and MDR-TB strains and the generation of novel therapeutic interventions to tackle the response of mycobacteria towards oxidative burden. The research has just only begun to assess the effects of a combination of therapies, including antitubercle antibiotics and agents targeting the oxidant : antioxidant axis, which may prove beneficial in eliminating the pathogen from the host in a shorter time frame.

## Figures and Tables

**Table 1:** Classification of drugs for the treatment of MDR-TB.

Second-line drug group	Drug type	Specific drugs
Group A	Fluoroquinolones	Levofloxacin Moxifloxacin Gatifloxacin

Group B	Second-line injectable agents	Amikacin Capreomycin Kanamycin Streptomycin

Group C	Other core second-line agents	Ethionamide/prothionamide Cycloserine/terizidone Linezolid Clofazimine

Group D	Group D1	Pyrazinamide Ethambutol High-dose isoniazid

Group D	Group D2	Bedaquiline Delamanid

Group D	Group D3	*p*-Aminosalicylic acid Imipenem-cilastatin Meropenem Amoxicillin-clavulanate Thioacetazone
